# Supplementary material for: Pregnant women’s daily patterns of well-being before and during the COVID-19 pandemic in Finland: Longitudinal monitoring through smartwatch technology
Source: PLoS One. 2021 Feb 3;16(2):e0246494. doi: 10.1371/journal.pone.0246494 (PMC7857616; doi:10.1371/journal.pone.0246494)
Supplement: Attachment — Submitted filename: ResponsestotheReviewers.docx [file pone.0246494.s001.docx]

**Responses to the Editor and the Reviewer**

*Journal Requirements:*

*When submitting your revision, we need you to address these additional requirements.*

*1. Please ensure that your manuscript meets PLOS ONE's style requirements, including those for file naming. The PLOS ONE style templates can be found at*

[*https://journals.plos.org/plosone/s/file?id=wjVg/PLOSOne_formatting_sample_main_body.pdf*](https://journals.plos.org/plosone/s/file?id=wjVg/PLOSOne_formatting_sample_main_body.pdf) *and*

[*https://journals.plos.org/plosone/s/file?id=ba62/PLOSOne_formatting_sample_title_authors_affiliations.pdf*](https://journals.plos.org/plosone/s/file?id=ba62/PLOSOne_formatting_sample_title_authors_affiliations.pdf)

RESPONSE: We have used the given templates and followed the PLOS ONE’s style requirements throughout all the files.

*2.**Please provide the following information in your Methods section regarding validation of the  weekly question asked related to the COVID-19.*

 RESPONSE: The weekly questions were developed for this study and therefore not validated. We have added this information in the Methods section.

*3. We note that you have indicated that data from this study are available upon request. PLOS only allows data to be available upon request if there are legal or ethical restrictions on sharing data publicly. For information on unacceptable data access restrictions, please see* [*http://journals.plos.org/plosone/s/data-availability#loc-unacceptable-data-access-restrictions*](http://journals.plos.org/plosone/s/data-availability#loc-unacceptable-data-access-restrictions)*.*

*In your revised cover letter, please address the following prompts:*

*a) If there are ethical or legal restrictions on sharing a de-identified data set, please explain them in detail (e.g., data contain potentially identifying or sensitive patient information) and who has imposed them (e.g., an ethics committee). Please also provide contact information for a data access committee, ethics committee, or other institutional body to which data requests may be sent.*

*b) If there are no restrictions, please upload the minimal anonymized data set necessary to replicate your study findings as either Supporting Information files or to a stable, public repository and provide us with the relevant URLs, DOIs, or accession numbers. Please see* [*http://www.bmj.com/content/340/bmj.c181.long*](http://www.bmj.com/content/340/bmj.c181.long) *for guidelines on how to de-identify and prepare clinical data for publication. For a list of acceptable repositories, please see* [*http://journals.plos.org/plosone/s/data-availability#loc-recommended-repositories*](http://journals.plos.org/plosone/s/data-availability#loc-recommended-repositories)*.*

*We will update your Data Availability statement on your behalf to reflect the information you provide.*

 RESPONSE: We have revised the cover letter accordingly.

*4. Please include captions for your Supporting Information files at the end of your manuscript, and update any in-text citations to match accordingly. Please see our Supporting Information guidelines for more information:* [*http://journals.plos.org/plosone/s/supporting-information*](about:blank)*.*

RESPONSE: We do not have any Supporting information files in our submission.

***Review Comments to the Author***

***Reviewer #1:*** *Pregnant women’s daily patterns of well-being before and during the COVID-19
pandemic in Finland: longitudinal monitoring through smartwatch technology

This study examines two cohorts of pregnant women (N=38), whose PPG, sleep and physical activity data were collected over four week periods before and during
national stay-at-home restrictions in response to the COVID-19 pandemic. Overall, the writing is straightforward and a rationale for the study is clear and relevant.*

RESPONSE: We would like to thank the Reviewer for the positive and constructive criticism provided. Our detailed responses to the questions and concerns below. The changes in the manuscript text are made with “track changes”.

*There are several points at which the authors could improve the clarity of the methods as well as their interpretation of the findings. It is unclear for what type of overall study these women were recruited and that could be made more apparent.*

RESPONSE: We have added more detailed description of the overall study where these women were recruited in the Study design paragraph.

*In terms of the device used to collect the data, it might be helpful to know whether this device has been validated or used for research purposes or to mention earlier than in the limitations section.*

RESPONSE: We have added a sentence about the validity of the smartwatch in the beginning of the Data collection paragraph. In addition, we modified the text regarding validity in the Limitations section.

*The authors also include both high and low risk women in this sample and report demographics and mean EPDS and anxiety scores. This has the potential to confound some meaningful data. First, the authors might add validity to their approach by showing statistically that these two groups do not differ in meaningful ways across demographics and clinical characteristics. They might also add these analyses to their outcomes, as there may be distinct differences in the overall stress of women who are high-risk and women who are low-risk in this sample and those differences may be observed in both HRV and sleep. If these groups are statistically no different, than these findings are valid. If not, there is an issue here of examining qualitatively distinct groups that are better off being examined separately. Alternately, the authors can explain their rationale in including all of these women in the same study via providing more context for the overall study (specifics that are aforementioned in this review).*

RESPONSE: We have added the background characteristics of both high-risk and low-risk groups separately in the Table 1. There were no differences between the groups except the gestational weeks during the study period since the recruitment of the participants in the high-risk group was initiated and completed earlier. However, we used the group also as an independent variable in the statistical models to confirm there were no differences between these groups.

*The authors find that SDNN increased across time despite the LF and LF/HF ratio increasing. These results are somewhat counter to one another, but the author discusses this as if there is a linear relationship between stress and HRV (starting in line 340). It might be more helpful to discuss this counterintuitive finding considering what aspects of autonomic functioning contribute to SDNN vs LF HRV ( see Shaffer & Ginsberg, 2017 for a broad but meaningful overview). In fact, HRV may become blunted or hypoactive in the face of acute stressors In this sample, it is possible that there may be other clinical factors at play (increases in depression or anxiety or event symptoms of PTSD) that might impact parameters of HRV in either the time or frequency domains. The authors do not control for any other factors related to HRV, including weight or physical health and it is incredibly difficult to draw conclusions related to HRV without controlling for these factors or holding other factors consistent, including time of day when the data was extracted.*

*The authors might do well to refer to major papers that discuss these recommended methods directly and include these potential confounders in their paper if they have them or address them as limitations if they do not (Laborde, Mosley & Thayer, 2017). While I agree that this article has merit, the oversight in terms of psychophysiological data analyses and interpretation is hard to ignore.*

*Laborde, S., Mosley, E., & Thayer, J. F. (2017). Heart rate variability and cardiac vagal tone in psychophysiological research–recommendations for experiment planning, data analysis, and data reporting. Frontiers in psychology, 8, 213.

Shaffer, F., & Ginsberg, J. P. (2017). An overview of heart rate variability metrics and norms. Frontiers in public health, 5, 258*

RESPONSE: Thank you for pointing this out. We have now modified the Discussion section in order to emphasize the challenges in interpreting the HRV results. We were not able to control all the confounding variables, however, we used only night-time data thus the day of the time was controlled. The Limitations section has also been modified to provide a clear picture of which parameters were controlled and which not. Further, we have added the papers the Reviewer suggested in the discussion and found them very relevant and useful for our manuscript.
